# Estimation of basic reproduction number of the Middle East respiratory syndrome coronavirus (MERS-CoV) during the outbreak in South Korea, 2015

**DOI:** 10.1186/s12938-017-0370-7

**Published:** 2017-06-13

**Authors:** Hyuk-Jun Chang

**Affiliations:** School of Electrical Engineering, Kookmin University, 77 Jeongneung-ro, Seongbuk-gu, 136-702 Seoul, Korea

**Keywords:** Basic reproduction number, Susceptible-infected-removed (SIR) model, Middle East respiratory syndrome (MERS), MERS coronavirus (MERS-CoV)

## Abstract

**Background:**

In South Korea, an outbreak of Middle East respiratory syndrome (MERS) occurred in 2015. It was the second largest MERS outbreak. As a result of the outbreak in South Korea, 186 infections were reported, and 36 patients died. At least 16,693 people were isolated with suspicious symptoms. This paper estimates the basic reproduction number of the MERS coronavirus (CoV), using data on the spread of MERS in South Korea.

**Methods:**

The basic reproduction number of an epidemic is defined as the average number of secondary cases that an infected subject produces over its infectious period in a susceptible and uninfected population. To estimate the basic reproduction number of the MERS-CoV, we employ data from the 2015 South Korea MERS outbreak and the susceptible-infected-removed (SIR) model, a mathematical model that uses a set of ordinary differential equations (ODEs).

**Results:**

We fit the model to the epidemic data of the South Korea outbreak minimizing the sum of the squared errors to identify model parameters. Also we derive the basic reproductive number as the terms of the parameters of the SIR model. Then we determine the basic reproduction number of the MERS-CoV in South Korea in 2015 as 8.0977. It is worth comparing with the basic reproductive number of the 2014 Ebola outbreak in West Africa including Guinea, Sierra Leone, and Liberia, which had values of 1.5–2.5.

**Conclusions:**

There was no intervention to control the infection in the early phase of the outbreak, thus the data used here provide the best conditions to evaluate the epidemic characteristics of MERS, such as the basic reproduction number. An evaluation of basic reproduction number using epidemic data could be problematic if there are stochastic fluctuations in the early phase of the outbreak, or if the report is not accurate and there is bias in the data. Such problems are not relevant to this study because the data used here were precisely reported and verified by Korea Hospital Association.

## Background

The Middle East respiratory syndrome (MERS) is caused by a coronavirus (CoV), the MERS-CoV. In Saudi Arabia, the first case of the disease was reported in 2012 [1]. The first case of MERS in the Republic of Korea was identified on 20 May 2015 [2]. A significant outbreak of MERS occurred in South Korea and lasted for almost three months, from May to July 2015.

The 2015 MERS spread in South Korea is the second largest outbreak recorded to date [3]. As a result of the outbreak in South Korea, 186 infections were reported, and 36 patients died. At least 16,693 people were isolated with suspicious symptoms [4]. This paper evaluates the basic reproduction number of MERS-CoV, using data from the 2015 South Korea outbreak.

The basic reproduction number (generally denoted as $R_0$) of an epidemic is defined as the average number of secondary cases that an infected subject produces over its infectious period in a susceptible and uninfected population [5–11]. It can estimate the growth rate of an infectious disease at the early stage of the outbreak, when most individuals are susceptible [12]. The basic reproduction number of an epidemic is useful for determining whether an outbreak of the disease will occur or not [13], and for analyzing epidemic properties of the disease further [14].

Note that $R_0$ is referred to as the basic reproductive (or reproduction) number (or ratio). The basic reproductive (or reproduction) rate is incorrect nomenclature because $R_0$ is a dimensionless number that is not related to any physical quantity corresponding to rate.

Based on [11], the reasons to estimate the basic reproduction number of an epidemic are summarized as follows: First, we can relatively evaluate the risk of the corresponding epidemic using $R_0$. In other words, we can compare the infectivity of the epidemic with others, already familiar to us. Second, the reproduction number can be evaluated multiple times, e.g., before (and after) an infection control measure intervention. To this end, it is needed to distinguish the reproduction number after control intervention from the basic reproductive number $R_0$, which is estimated before the intervention. Now we refer to the reproduction number after control intervention as the effective reproduction number, denoted by $R_{\text {eff}}$. Then we can compare $R_{\text {eff}}$ **with** $R_0$, and we can evaluate the efficacy of a control measure quantitatively based on $R_{\text {eff}}$. By doing so, eventually we can determine how to apply control intervention to reduce $R_{\text {eff}}$ to less than one. If $R_{\text {eff}} < 1$, it is concluded that the control intervention works effectively, and that the outbreak will eventually be controlled by reducing the reproduction number to less than the threshold level, 1.

In this paper, to estimate the basic reproduction number of the MERS-CoV, we employ data from the 2015 South Korea MERS outbreak and the susceptible-infected-removed (SIR) model [13, 15, 16], a mathematical model that uses a set of ordinary differential equations (ODEs). Because the availability of epidemic data is limited, we usually employ non-structured deterministic models to evaluate $R_0$ [11].

Based on the data reported from the 2015 South Korea outbreak of MERS, we evaluate the basic reproductive number of the virus, MERS-CoV. We fit the model to epidemic data from the South Korea outbreak, and identify model parameters and the basic reproduction number, $R_0$. Note that other epidemiological parameters, such as incubation period and serial interval, have been discussed in [17] for the outbreak.

Preliminary work relating to this paper is presented in [18]. This paper includes an analysis of the derivation of the basic reproduction number, careful screening of the reported data, and a sophisticated approach using the sum of the squared errors to evaluate the basic reproduction number precisely.

A number of papers and books have been dedicated to the study of $R_0$ for other infectious diseases. A few of them are as follows: see [19] for the $R_0$ of severe acute respiratory syndrome(SARS), [20] for the $R_0$ of influenza, [21, 22] for the $R_0$ of Ebola, and [23] for the $R_0$ of malaria.

Although some literature on the study of $R_0$ of the MERS-CoV has been reported such as [12, 24], the MERS outbreak in South Korea is unique [25]. MERS spread almost naturally without any intervention in the early stage, and the Korean government did not respond appropriately [3, 25]. The list of medical facilities involved was not even announced to public. Ironically, it is the ideal condition to fit a mathematical model to the clinical epidemic data and to evaluate epidemic properties of the MERS-CoV, including the basic reproduction number.

The paper is organized as follows: In “Methods” section, a mathematical model, which comprises a set of ordinary differential equations, is introduced, and an estimation method is discussed for the basic reproductive number. “Results” section describes the evaluation result, providing further discussion. “Conclusions” section concludes the paper by suggesting additional research.

## Methods

In this section, we briefly discuss the method employed in this paper, including a definition of the basic reproduction number, and we introduce the SIR model.

### Basic reproduction number

The basic reproduction number is defined as the number of secondary cases that one infected primary subject causes on average in an uninfected and totally susceptible population, over the infectious period [8–10]. Based on this definition, we obtain a mathematical description of $R_0$ via the so-called ‘survival function’ [7, 11].

Considering a large population, this description is given by

1 $$\begin{aligned} R_0 = \int _0^\infty {b(a)F(a)}da \end{aligned}$$

where *F*(*a*) is the survival function describing the probability of a newly infected patient will be infectious at least to time *a*, and *b*(*a*) is the average number of infected subjects whom one infected patient will produce per unit time at time *a* [7, 11]. The notation of (1) follows the usage therein.

Formula (1) is derived from the $R_0$ definition and can thus be used for any mathematical model, not just models given by ODEs. However, it requires explicit expressions for $F(\cdot )$ and $b(\cdot )$, which are functions of time. This paper employs the SIR model described by ODEs that is introduced in the next section. Because the model does not provide explicit descriptions for $F(\cdot )$ and $b(\cdot )$, we use an alternative expression for $R_0$, derived from the SIR model.

### SIR model

To investigate the spread dynamics of epidemics, several nonlinear mathematical models have been studied. We employ one of the models, the SIR model that is described by

2 $$\begin{aligned} \dot{S}&= - \beta S I, \nonumber \\ \dot{I}&= \beta S I - \nu I, \nonumber \\ \dot{R}&= \nu I, \end{aligned}$$

where the states *S*, *I*, and *R* correspond to the number of susceptible people, the number of infected, and the number of removed, respectively. Note that state *R* includes both deceased and recovered patients. For each subject group of *S*, *I*, and *R*, we assume that the properties of the subjects (for example, infectiveness, susceptibility, and so forth) are homogeneous. The parameter $\beta$ is the disease transmission rate and the parameter $\nu$ is the removed rate. Note that both parameters are positive real.

The notation of (2) follows that used in [16]. Table 1 presents descriptions of system parameters and state variables for model (2). Parameters $\kappa$ and $\tau$ are discussed further in the “Results” section. For a further explanation of the SIR model, see [13, 15, 16].

**Table 1:** Descriptions of system parameters and state variables of model (2) and the equation (3)

State variables, parameters	Descriptions
*S*	Number of susceptible subjects
*I*	Number of infected subjects
*R*	Number of removed subjects
$\beta$	Disease transmission rate
$\nu$	Removed rate
$\tau$	Transmissibility of the infection
$\kappa$	Number of transmittable contacts by infected patient per unit time

Any term related to birth and death in the population that is not caused by MERS is not included in the model (2). The dynamics of the disease (e.g., infection or recovery) is assumed to be significantly faster than that of birth and death in the population. Generally, epidemic models such as SIR do not include birth and death because zero net change of the population is assumed. If we model an infectious disease with comparatively slow dynamics (e.g., an endemic disease), we must consider dynamic terms describing birth and death.

The system parameters, which are all rates, are positive and real in the model (2). Because of the definition of $R_0$, an alternative description for $R_0$ can be derived from model (2):

3 $$\begin{aligned} R_0 = \kappa \tau d, \end{aligned}$$

where *d* is the infection duration, $\kappa$ is the transmittable contact number by one infected subject per one unit time, and $\tau$ is the *transmissibility* of the infectious disease, which corresponds to the probability of infection per one contact between an infected patient and a susceptible individual.

It is notable that the removed rate is reciprocal to the infection duration by the assumption of constant rates for the SIR model (2):

$$\begin{aligned} d = \frac{1}{\nu }. \end{aligned}$$

## Results

In Table 2, the history of the MERS-CoV spread status is presented. The Ministry of Health and Welfare, Korea officially announced the data. “Infected” represents the accumulated number of infected patients. “Deceased” is the number of dead subjects. “Recovered” is the number of individuals returning to healthy status. All entries in the table are as of the “Date”. The number of infected patients includes both removed and recovered patients.

Table 2 shows the MERS-CoV spread data for only the initial phase of the outbreak, i.e., from 20 May 2015 to 12 June 2015.

On 7 June 2015, the South Korean government disclosed to the public the list of all hospitals exposed to MERS-CoV, with the dates and duration of exposure [4]. This is the first intervention of the government to control the spread. Before this date, there was no control action that could affect estimation of the basic reproduction number of MERS-CoV.

The incubation period of MERS-CoV that can range from 2 to 14 days, is 5 days on average [26]. Thus, we use reported data up to 12 June 2015.

**Table 2:** Accumulated MERS-CoV patients in Korea, 2015 [4]

Date	Infected	Deceased	Recovered
20 May	2	0	0
21 May	3	0	0
22 May	3	0	0
23 May	3	0	0
24 May	3	0	0
25 May	3	0	0
26 May	5	0	0
27 May	5	0	0
28 May	7	0	0
29 May	13	0	0
30 May	15	0	0
31 May	18	0	0
1 June	25	1	0
2 June	30	1	0
3 June	30	3	0
4 June	36	4	0
5 June	42	5	1
6 June	64	5	1
7 June	87	5	1
8 June	95	7	2
9 June	108	7	3
10 June	122	9	4
11 June	126	10	7
12 June	138	13	9

The total population size is denoted by *N*(*t*):

$$\begin{aligned} N(t):=S(t)+I(t)+R(t). \end{aligned}$$

It can be seen that

$$\begin{aligned} \dot{N}(t)&=\dot{S}(t) + \dot{I}(t) + \dot{R}(t)\\&= 0 \end{aligned}$$

from the SIR model (2) (see the left-hand sides of the SIR model equations and their sum). This implies that *N*(*t*) can be assumed to be constant for the SIR model (2), *i.e.,* $N(t)=N(0):=N$. With this assumption, we consider the state *S*(*t*) as $N - I(t) - R(t)$. Then, we can describe the SIR model (2) as a two-dimensional model.

Considering the magnitude of the numbers in Table 2, it is assumed that

4 $$\begin{aligned} N \cong S. \end{aligned}$$

Compared to the total size of the population, the number of outbreak cases is small. The *N* of South Korea is now known to be over 51 million. If the number of outbreak cases is much smaller than the total size of the population, the number *N* does not need to be exact to estimate system parameters of the SIR model (2) [21, 22].

From [16],

$$\begin{aligned} \beta = \frac{\kappa \tau }{N}, \end{aligned}$$

where $\tau$ is the transmissibility of the infectious disease and $\kappa$ is the transmittable contact number by one infected subject per one unit time.

Then, the model where the dimension is reduced by assumption (4) is

5 $$\begin{aligned} \dot{I}&= \kappa \tau I - \nu I, \nonumber \\ \dot{R}&= \nu I. \end{aligned}$$

The initial state for the SIR model is provided by Table 2, given by

6 $$\begin{aligned}{}[I(0), R(0)]=[2, 0]. \end{aligned}$$

Now, we only need to know the parameter pair, ($\kappa \tau$, $\nu$), to solve model (5) numerically. To solve the mathematical model and to evaluate the basic reproduction number of the model, we do not need to know each $\kappa$ and $\tau$ necessarily, but we can use the value of the product, i.e., $\kappa \tau$.

We search for the parameter pair ($\kappa \tau$, $\nu$) such that can respond appropriately with the data in Table 2. To evaluate how closely system response is fitted to the data in Table 2, we employ a quantitative measure, *the sum of the squared errors*. Once we obtain the optimal values for the parameters with respect to this measure, we can estimate the basic reproduction number as described in the “Methods” section.

We define the measure $f_{E}(\cdot , \cdot )$ as

7 $$\begin{aligned} f_{E}(\kappa _i \tau _i, \nu _i) = \sqrt{\sum _{k=0}^{n} \left( \left( I_i(k) - I_T(k) \right) ^2 + \left( R_i(k) - R_T(k) \right) ^2 \right) } \end{aligned}$$

where $n = 23$ and *k* corresponds to the date. For example, $k = 0$ and $k = 23$ indicate the dates 20 May 2015 and 12 June 2015, respectively. $I_i(\cdot )$ and $R_i(\cdot )$ are from the simulation result of model (5) with the initial condition (6) and the corresponding parameters, i.e., the function arguments ($\kappa _i \tau _i$, $\nu _i$). $R_T(k)$ is the sum of the values of the “Deceased” and “Recovered” at *k* in Table 2. $I_T(k)$ is the value, $R_T(k)$ subtracted from the value of the “Infected” at *k*. See Table 3. Based on the data of Table 2, $I_T(k)$ and $R_T(k)$ along with *k* are shown in Table 3.

**Table 3:** $I_T(k)$ and $R_T(k)$ of function (7), derived from Table 2

*k*	$I_T(k)$	$R_T(k)$
0	2	0
1	3	0
2	3	0
3	3	0
4	3	0
5	3	0
6	5	0
7	5	0
8	7	0
9	13	0
10	15	0
11	18	0
12	24	1
13	29	1
14	27	3
15	32	4
16	36	6
17	58	6
18	81	6
19	86	9
20	98	10
21	109	13
22	109	17
23	116	22

To compare the quantitative measures for each pair of parameters, we consider the plane, i.e., 2-dimensional space, of the parameters, ($\kappa \tau$, $\nu$). We can obtain a surface in 3-dimensional space by plotting the corresponding measure as the value along the third axis.

**Fig. 1:**
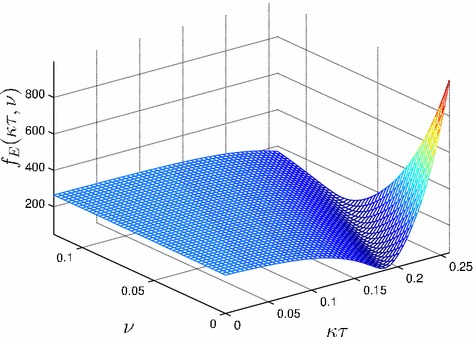
Plot of $f_{E}(\kappa \tau , \nu )$ of (7) on the plane of ($\kappa \tau$, $\nu$). $f_{E}(\cdot , \cdot )$ is a quantitative measure, the sum of the squared errors, that describes how the system response of model (5) with corresponding parameters is close to the data in Table 2. The ranges of $\kappa \tau$ and $\nu$ in this figure are [0.00, 0.26] and [0.00, 0.12], respectively

We explore the plane for wide ranges of parameters, and one of the results is shown in Fig. 1. This figure can help show the relation between $f_{E}$ and the system parameters. To effectively capture the characteristics of the measure, the ranges of $\kappa \tau$ and $\nu$ in the figure are [0.00, 0.26] and [0.00, 0.12], respectively.

**Fig. 2:**
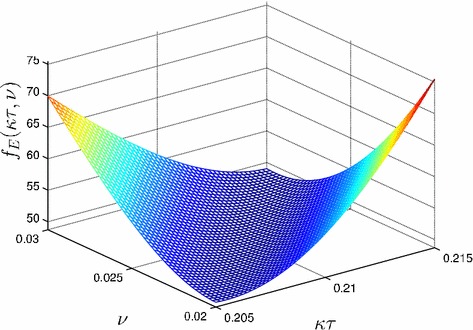
Plot of $f_{E}(\kappa \tau , \nu )$ of (7) on the plane of ($\kappa \tau$, $\nu$). Compared to Fig.  1, the ranges of $\kappa \tau$ and $\nu$ in this figure are [0.2050, 0.2150] and [0.0200, 0.0300], respectively, and they can be used to obtain precise values of system parameters to minimize the quantitative measure $f_{E}$

Based on characteristics shown in Fig.  1, we search for precise values for $\kappa \tau$ and $\nu$ to minimize the measure, $f_{E}$. Consider the parameter plane with the range [0.2050, 0.2150] of $\kappa \tau$ and with the range [0.0200, 0.0300] of $\nu$ in Fig.  2. Then we can identify the precise values for $\kappa \tau$ and $\nu$ using Fig.  2. The system parameters are $\kappa \tau = 0.21153$ and $\nu = 0.026122$ evaluated by Fig.  2 to minimise the quantitative measure $f_{E}$

The function $f_E(\cdot , \cdot )$ describes the sum of squared error between the outbreak data of Table 2 and the simulation result of model (5) with the initial condition (6) and the function arguments. Thus, if we find the parameters minimizing the function (7), then these parameters can be considered to correspond to the case of Table 2.

**Fig. 3:**
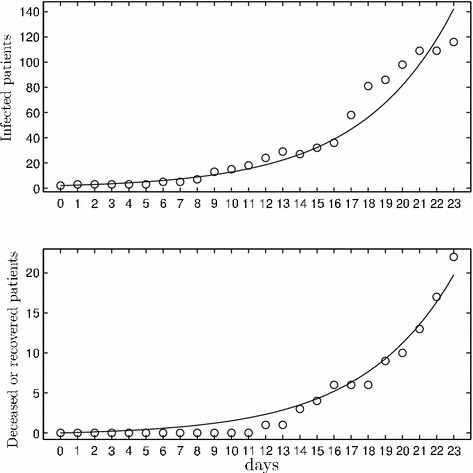
Data presented in Table 2 and the state trajectories of model (5) with the estimated parameters. In the* top panel*, the* circle marks* plot the number of “Infected” patients from Table 2, and the* solid line* depicts the transition of state *I* of model (5). In the* bottom panel*, the* circle marks* indicate the sum of the numbers of “Deceased” and “Recovered” patients, and the* solid line* depicts the transition of state *R* of the model

Figure 3 shows the data presented in Table 2 and the state trajectories of model (5) with the parameter values obtained from Fig.  2. The top panel of Fig.  3 shows infected patients, and the bottom panel shows deceased or recovered patients. In both panels, the circle marks and the solid line display patient numbers based on Table 2 and the state transition of model (5), respectively.

We derive the *basic reproductive number* for the SIR model (2) as

8 $$\begin{aligned} R_0=\frac{\kappa \tau }{\nu } \end{aligned}$$

in the “Methods” section, based on [16]. Equation (8) can be obtained alternatively by using the next generator approach [6, 8, 11, 27] to model (5).

We determine the $R_0$ of the MERS-CoV in South Korea in 2015 as 8.0977 (*i.e.,* 0.21153/0.026122). It is worth comparing with $R_0$ of the 2014 Ebola outbreak, which had values of 1.5–2.5 [21].

## Conclusions

In this paper, we evaluated the basic reproduction number of the MERS-CoV outbreak that occurred in 2015 in South Korea, using officially reported data. We employed a mathematical dynamic model, the SIR model. We first fit the response of the SIR model to the epidemic curve data reported from the MERS outbreak. Then, we identified the system parameters of the model to estimate the basic reproduction number.

Because there was no intervention to control the infection in the early phase of the outbreak, the data used here provide the best conditions to evaluate the epidemic characteristics of MERS, such as the basic reproduction number. An evaluation of $R_0$ using epidemic data could be problematic if there are stochastic fluctuations in the early phase of the outbreak, or if the report is not accurate and there is bias in the data [11]. Such problems are not relevant to this study because the data used here were precisely reported and verified by [4].

We conclude this paper with the following discussion on future work to overcome the limitations of research, derived from assumptions in the paper.

### Further research direction

- Behind the SIR model (2), there are several strong assumptions, one of which is a zero latent period, i.e., the incubation period is zero. This implies that a patient becomes infectious immediately after infection. However, the incubation phase occurs during the course of the MERS outbreak. To address this weak point, in future work we could consider the 4-dimensional SEIR (i.e., susceptible-exposed-infectious-removed) model, which has been employed in [28] to study Ebola epidemic model. The additional state in the SEIR model can help us deal with the latent period.
- In this paper, we considered the epidemic curve data in [4] only from the early stage of the 2015 MERS outbreak in South Korea, where there was no intervention to control the spread. Accordingly, we evaluated $R_0$ based on the data. In future work, we will also consider the epidemic data in [4] from the later (or closing) stage of the MERS spread in South Korea in 2015, so we can estimate the effective production number (i.e., $R_\mathrm{eff}$), which is the production number resulting from interventions, such as education, quarantine, and the tracing of contacts by infected patients. By doing so, we can evaluate the effectiveness of each control measure on the spread of the infectious disease[29]. Eventually, such evaluation could help us improve public health policy.

## References

[1] World Health Organization: Fact Sheet 401: Middle East respiratory syndrome coronavirus (MERS-CoV). 2015. http://www.who.int/mediacentre/factsheets/mers-cov/en/. Accessed 8 May 2017.

[2] Su S, Wong G, Liu Y, Gao GF, Li S, Bi Y (2015). MERS in South Korea and China: a potential outbreak threat?. Lancet.

[3] Hyun MH, Park Y (2015). Re: Why the panic? South Korea’s MERS response questioned. BMJ.

[4] Korea Hospital Association MERS. White Paper by Korea Hospital Association. Korea Hospital Association, Seoul; 2015.

[5] Anderson RM, May RM (1992). Infectious diseases of humans: dynamics and control.

[6] Diekmann O, Heesterbeek JAP, Metz JAJ (1990). On the definition and the computation of the basic reproduction ratio $${R}_0$$ in models for infectious diseases in heterogeneous populations. J Math Biol.

[7] Heesterbeek JAP, Dietz K (1996). The concept of $$R_0$$ in epidemic theory. Stat Neerl.

[8] Diekmann O, Heesterbeek JAP (2000). Mathematical epidemiology of infectious diseases: model building. Analysis and interpretation.

[9] van den Driessche P, Watmough J (2002). Reproduction numbers and sub-threshold endemic equilibria for compartmental models of disease transmission. Math Biosci.

[10] Heesterbeek JAP (2002). A brief history of $$R_0$$ and a recipe for its calculation. Acta Biotheor.

[11] Heffernan JM, Smith RJ, Wahl LM (2005). Perspectives on the basic reproductive ratio. J R Soc Interface.

[12] Chowell G, Blumberg S, Simonsen L, Miller MA, Viboud C (2014). Synthesizing data and models for the spread of MERS-CoV, 2013: key role of index cases and hospital transmission. Epidemics.

[13] DiStefano J (2015). Dynamic systems biology modeling and simulation.

[14] Yashima K, Sasaki A (2016). Spotting epidemic keystones by $$R_0$$ sensitivity analysis: high-risk stations in the Tokyo metropolitan area. PLOS ONE.

[15] Anderson RM (1991). Discussion: the Kermack-McKendrick epidemic threshold theorem. Bull Math Biol.

[16] Weiss H (2013). The SIR model and the foundations of public health. MATerials MATemàtics.

[17] Cowling BJ, Park M, Fang VJ, Wu P, Leung GM, Wu JT (2015). Preliminary epidemiologic assessment of MERS-CoV outbreak in South Korea, May to June 2015. Euro Surveill..

[18] Chang H. Evaluation of the basic reproduction number of MERS-CoV during the 2015 outbreak in South Korea. In: Proceedings of 16th international conference on control, automation and systems. 2016. p. 981–984

[19] Wallinga J, Teunis P (2004). Different epidemic curves for severe acute respiratory syndrome reveal similar impacts of control measures. Am J Epidemiol.

[20] Mills CE, Robins JM, Lipsitch M (2004). Transmissibility of 1918 pandemic influenza. Nature.

[21] Althaus CL (2014). Estimating the reproduction number of ebola virus (EBOV) during the 2014 outbreak in west africa. PLOS Curr Outbreaks.

[22] Chowell G, Hengartner NW, Castillo-Chavez C, Fenimore PW, Hyman JM (2004). The basic reproductive number of Ebola and the effects of public health measures: the cases of Congo and Uganda. J Theor Biol.

[23] Smith DL, McKenzie FE, Snow RW, Hay SI (2007). Revisiting the basic reproductive number for malaria and its implications for malaria control. PLoS Biol.

[24] Ejima K, Aihara K, Nishiura H (2014). Probabilistic differential diagnosis of middle east respiratory syndrome (MERS) using the time from immigration to illness onset among imported cases. J Theor Biol.

[25] Ha K-M (2016). A lesson learned from the MERS outbreak in South Korea in 2015. J Hosp Infect.

[26] Assiri A, McGeer A, Perl TM, Price CS, Al Rabeeah AA, Cummings DAT, Alabdullatif ZN, Assad M, Almulhim A, Makhdoom H, Madani H, Alhakeem R, Al-Tawfiq JA, Cotten M, Watson SJ, Kellam P, Zumla AI, Memish ZA (2013). Hospital outbreak of middle east respiratory syndrome coronavirus. N Engl J Med.

[27] Area I, Losada J, Ndaïrou F, Nieto JJ, Tcheutia DD. Mathematical modeling of 2014 Ebola outbreak. Math Methods Appl Sci. 2015. doi:10.1002/mma.3794.

[28] Area I, Batarfi H, Losada J, Nieto JJ, Shammakh W, Torres Á (2015). On a fractional order Ebola epidemic model. Adv Differ Equ.

[29] Nishiura H, Ejima K, Mizumoto K, Nakaoka S, Inaba H, Imoto S, Yamaguchi R, Saito M (2014). Cost-effective length and timing of school closure during an influenza pandemic depend on the severity. Theor Biol Med Model.

